# *N*-Acetylcysteine Enhances the Recovery of Ischemic Limb in Type-2 Diabetic Mice

**DOI:** 10.3390/antiox11061097

**Published:** 2022-05-31

**Authors:** Qiang Zhu, Xuanyou Liu, Qingyi Zhu, Zehao Liu, Chunlin Yang, Hao Wu, Linfang Zhang, Xiujuan Xia, Meifang Wang, Hong Hao, Yuqi Cui, Guangsen Zhang, Michael A. Hill, Gregory C. Flaker, Shenghua Zhou, Zhenguo Liu

**Affiliations:** 1 Center for Precision Medicine, Department of Medicine, Division of Cardiovascular Medicine, University of Missouri School of Medicine, Columbia, MO 65212, USA; zhuqia@health.missouri.edu (Q.Z.); liuxua@health.missouri.edu (X.L.); zhuqingyi@csu.edu.cn (Q.Z.); zehaoliu@csu.edu.cn (Z.L.); yangchunl@health.missouri.edu (C.Y.); hw5mg@missouri.edu (H.W.); zhanglinf@missouri.edu (L.Z.); 168301005@csu.edu.cn (X.X.); wangmei@health.missouri.edu (M.W.); haoho@health.missouri.edu (H.H.); cuiyu@health.missouri.edu (Y.C.); flakerg@health.missouri.edu (G.C.F.); 2 Department of Cardiology, Second Xiangya Hospital, Central South University, Changsha 410011, China; zhoushenghua@csu.edu.cn; 3 Institute of Molecular Hematopathy, Second Xiangya Hospital, Central South University, Changsha 410011, China; zhangguangsen@csu.edu.cn; 4 Dalton Cardiovascular Research Center, University of Missouri, Columbia, MO 65211, USA; hillmi@missouri.edu

**Keywords:** reactive oxygen species, oxidative stress, *N*-acetylcysteine, type-2 diabetes, limb ischemia, insulin resistance

## Abstract

Critical limb ischemia (CLI) is a severe complication of diabetes mellitus that occurs without effective therapy. Excessive reactive oxygen species (ROS) production and oxidative stress play critical roles in the development of diabetic cardiovascular complications. *N*-acetylcysteine (NAC) reduces ischemia-induced ROS production. The present study aimed to investigate the effect of NAC on the recovery of ischemic limb in an experimental model of type-2 diabetes. TALLYHO/JngJ diabetic and SWR/J non-diabetic mice were used for developing a CLI model. For NAC treatment, mice received NAC (1 mg/mL) in their drinking water for 24 h before initiating CLI, and continuously for the duration of the experiment. Blood flow, mechanical function, histology, expression of antioxidant enzymes including superoxide dismutase (SOD)-1, SOD-3, glutathione peroxidase (Gpx)-1, catalase, and phosphorylated insulin receptor substrate (IRS)-1, Akt, and eNOS in ischemic limb were evaluated in vivo or ex vivo. Body weight, blood glucose, plasma advanced glycation end-products (AGEs), plasma insulin, insulin resistance index, and plasma TNF-a were also evaluated during the experiment. NAC treatment effectively attenuated ROS production with preserved expressions of SOD-1, Gpx-1, catalase, phosphorylated Akt, and eNOS, and enhanced the recovery of blood flow and function of the diabetic ischemic limb. NAC treatment also significantly decreased the levels of phosphorylated IRS-1 (Ser307) expression and plasma TNF-α in diabetic mice without significant changes in blood glucose and AGEs levels. In conclusion, NAC treatment enhanced the recovery of blood flow and mechanical function in ischemic limbs in T2D mice in association with improved tissue redox/inflammatory status and insulin resistance.

## 1. Introduction

Diabetes mellitus (DM) increases the risk of developing severe peripheral artery disease (PAD) with critical limb ischemia (CLI) and chronic ulcers [1]. Due to poor healing, patients with diabetes and CLI usually have poor outcomes, often requiring limb amputation and accounting for about 60% of all non-traumatic amputations in the U.S. [2]. After a two-decade decline in diabetes-related nontraumatic lower-extremity amputation, recent data show an increase in amputation, particularly in young and middle-aged adults [3]. In addition, major randomized clinical trials have shown that blood glucose-lowering or anti-hypertensive therapies have very limited effects on reducing macrovascular complications, and no benefit on amputation rates in diabetic patients [4]. These data highlight an urgent need for alternate and effective approaches that are aimed at promoting the recovery of the ischemic diabetic limb to reduce lower limb amputation in DM.

Reactive oxygen species (ROS) production and oxidative stress are increased in DM [5,6], and excessive oxidative stress contributes to the development of diabetic complications, including cardiovascular diseases, nephropathy, and retinopathy [7,8,9]. High levels of ROS contribute to a variety of pathological conditions, through reduced nitric oxide bioavailability, impairment of vascular function, and other endothelial cell phenotypic abnormalities [10]. Several sources of excessive ROS production have been noted in DM including metabolic abnormalities, which cause mitochondrial superoxide overproduction in endothelial cells of both large and small vessels, as well as in the myocardium [7]. N-acetylcysteine (NAC) is an FDA-approved drug with an established safety record which readily enters cells and increases intracellular levels of the antioxidant glutathione. The sulfhydryl group within the NAC molecule can also directly scavenge ROS and modulate the redox state both extracellularly and intracellularly [11]. Importantly, NAC has been reported to effectively decrease ischemia-induced ROS production both in vitro and in vivo in a few important organ systems including heart, lung, kidney, and the neurological system [12,13,14,15,16]. However, it is not clear if NAC is able to improve inflammatory status and enhance recovery of the ischemic limb, thereby being an approach for preventing ischemia-related lower-extremity amputation in diabetes.

Tumor necrosis factor α (TNF-α) is a potent mediator of inflammation, and serum TNF-α levels are significantly increased in DM, and contribute to increased levels of oxidative stress and insulin resistance [17]. The present study was designed to test the hypothesis that NAC suppresses ROS production, and improves redox/inflammatory status and insulin resistance, thus leading to improved blood flow recovery and limb function following ischemia in Type-2 diabetes (T2D). There were four objectives: (1) to determine if NAC could inhibit excessive ROS production in an experimental model of T2D; (2) to evaluate the effect of NAC on circulatory and functional recovery of the diabetic ischemic limb; (3) to evaluate the effect of NAC on plasma TNF-α levels, expressions of antioxidant enzymes, and phosphorylation of IRS-1 (Ser307), Akt (Ser473), and eNOS (Ser1177) in the diabetic ischemic limb; and (4) to define the metabolic effects of NAC in diabetic mice by evaluating the changes in body weight, blood glucose, plasma insulin, insulin resistance index, and plasma AGEs. The TALLYHO T2D mouse model was used since 90–95% of patients with diabetes have T2D [18].

## 2. Materials and Methods

### 2.1. Animals

All animal experiments were performed in compliance with the “Guide for the Care and Use of Laboratory Animals of US National Institutes of Health”. The experimental protocols were reviewed and approved by the Institutional Animal Care and Use Committee of the University of Missouri-Columbia (Protocol #10118). TALLYHO/JngJ mice (male, 16–20 weeks old, Jackson Laboratory, Bar Harbor, ME, USA) were used as a mouse Type-2 diabetic model, with SWR/J mice (male, 16–20 weeks old, Jackson Laboratory, Bar Harbor, ME, USA) serving as non-diabetic controls. SWR/J mice were used as the control for TALLYHO/JngJ mice since the two strains share 86.8% of their genotype and 67.1% of their haplotype based on TALLYHO/JngJ single nucleotide polymorphism (SNP) alignment with Swiss family strains [19,20].

### 2.2. Measurement of Body Weight, Blood Glucose, Plasma AGEs, and Insulin

To confirm the suitability of the T2D model, body weights, and blood glucose were measured using a weight scale and portable blood glucose meter (AimStrip, San Antonio, TX, USA) before and on days 14 and 21 after implementation of CLI (as described below). Blood glucose, plasma AGEs, and insulin levels were determined after overnight fasting and in the non-fasting state. AGEs were determined using a mouse AGE ELISA Kit (Biotang, Catalog# M7827, Lexington, MA, USA), and insulin from an ultra-sensitive mouse insulin ELISA Kit (Crystal Chem, Catalog# 90080, Elk Grove Village, IL, USA), together with a microplate reader (Biotek, Winooski, VT, USA). Insulin resistance (IR) was evaluated according to the homeostatic model assessment of insulin resistance (HOMA-IR). HOMA-IR = Fasting blood glucose level (mg/dL) * fasting insulin level (μIU/mL)/405 (μIU/mL = ng/mL × 21.2) [21,22].

### 2.3. Creation of CLI Model and Assessment of Blood Perfusion and Function

Mice were divided into 3 groups with 10 mice in each group: non-diabetic SWR/J mice control group (SWR), diabetic TALLYHO/JngJ mice group (TH), and diabetic TALLYHO/JngJ mice with NAC treatment group (TH & NAC). For the CLI model, unilateral hind limb ischemia was accomplished by ligation and transection of right femoral artery (at the site between the origin of the lateral caudal femoral and superficial epigastric arteries and genu artery) after a 2-mm incision with minimal tissue disturbance as described [23]. All the surgical procedures were conducted under air anesthesia (1.25% isoflurane) and constant temperature (37 ± 0.5 °C). For mice undergoing NAC treatment, NAC (Sigma-Aldrich, St. Louis, US) was added to drinking water (1 mg/mL) for 24 h before creation of CLI, and continuously throughout the duration of the experiment, based on our previous studies [16,24,25] and those of others [26,27]. Based on the amount of water consumed by the mice, the dose of NAC was estimated to be 0.5–1.0 g/kg/day.

Laser Doppler perfusion imaging (LDPI) was used to access blood perfusion in the ischemic limb with the normal limb acting as control (recovery of blood perfusion as reflected by the ratio of ischemic limb blood perfusion/normal limb blood perfusion) before ligation, 30 min, on days 3, 7, 14, and 21 after ligation under air anesthesia and constant temperature as described above. To evaluate the functional recovery of the ischemic limb, a swimming endurance test was performed as described [28]. Swimming time was determined before ligation, and on days 14 and 21 after ligation. A semi-quantitative assessment of ambulatory impairment and limitation of ischemic limb (modified clinical standard score) was conducted to assess the function of the ischemic limb at the same timepoints. The recovery index of the ischemic limb was as follows: 0: flexing the toes to resist gentle traction on the tail, 1: plantar flexion, 2: no dragging but no plantar flexion, and 3: foot dragging as described [29,30]. The number of normal, toe gangrene, and lower-extremity amputation in ischemic limb in each group were collected and classified at day 21.

### 2.4. CD31 Immunofluorescent Staining and DHE Staining for ROS Production

For analysis of CD31 immunofluorescence, the gastrocnemius muscle tissue of the ischemic limb was harvested at day 21 after ischemia. Frozen sections of the ischemic gastrocnemius muscle (6 μm) were sequentially fixed with 4% paraformaldehyde for 10 min, washed with PBS × 3, and blocked with 5% BSA for 30 min at room temperature. These preparations were then incubated with AF 594 anti-mouse CD31 immunofluorescent antibody (1:400, Biolegend, Catalog# 102432, San Diego, CA, USA) at 4 °C overnight. After three washes with PBS, the preparations were incubated with 4′,6-diamidino-2-phenylindole (DAPI) for 10 min at room temperature, and then washed three times for examination using a laser confocal microscope. Capillary density was quantified using ImageJ software. Multiple sections across the length of the muscle were obtained for each sample, and 2 sections were randomly selected for each sample for each of the histological classifications (CD31^+^ capillary density, DHE fluorescence, H&E staining, and Masson’s Trichrome staining, see below). From each section, 3 independent fields were examined with a laser confocal microscope or an inverted light microscope.

Detection of in situ O_2_^−^ formation was performed using the fluorescent dye dihydroethidium (DHE) as described [31]. Briefly, frozen sections of ischemic limb muscle were fixed with 4% paraformaldehyde for 10 min, and incubated with DHE (1:1000, Invitrogen, D1168) at room temperature for a further 15 min. The preparations were then washed three times with PBS, incubated with DAPI as described above, and visualized with a laser confocal microscope and quantified using ImageJ software.

### 2.5. Histological Analysis for Muscle Structure and Tissue Fibrosis

To assess the morphology and structure of muscle tissue in the ischemic limb, frozen muscle sections were stained using an H&E staining kit (For frozen sections, Thermo Scientific, Waltham, MA, USA) according to the manufacturer’s instructions. For analysis of tissue fibrosis, the gastrocnemius muscle of the ischemic limb was harvested at day 21 after induction of CLI, and fixed with 10% formalin overnight at room temperature. Paraffin sections (6 μm) were stained with Masson’s trichrome (Thermo Scientific, Waltham, MA, USA) as per manufacturer’s instruction after deparaffinization and rehydration. The preparations were examined from 3 random fields for each section using an inverted light microscope. The fibrosis area was determined with ImageJ software and presented as the ratio of fibrosis/total area.

### 2.6. Western Blot Assay and Measurement of Plasma TNF-α

The gastrocnemius muscle of the ischemic limb was harvested, homogenized, and lysed by ultrasound for western blot analysis as described [32]. Tissue lysates were loaded and separated on 10% SDS-PAGE gels, and then transferred onto 0.45 μm polyvinylidene difluoride (PVDF) membranes. After blocking with 5% milk in 1 × TBST buffer, the preparations were incubated with primary antibodies at 4 °C overnight, followed by incubation with corresponding second antibodies at room temperature for 1 h. The primary antibodies were: Akt (1:1000, Cell Signaling Technology, 9272, Danvers, MA, USA), p-Akt (Ser473, 1:1000, Cell Signaling Technology, 4060), eNOS (1:1000, Cell Signaling Technology, 9572), p-eNOS (Ser1177, 1:1000, Cell Signaling Technology, 9571), IRS-1 (1:1000, Cell Signaling Technology, 2382), p-IRS-1 (Ser307, 1:1000, Cell Signaling Technology, 2381), SOD-1 (1:1000; Invitrogen, MA1-105, Waltham, MA, USA), SOD-3 (1:1000; Invitrogen, PA5-93329), Gpx-1 (1:500; Invitrogen, 702762), Catalase (1:1000; Cell Signaling Technology, 14097S), VEGFR2 (1:1000; Cell Signaling Technology, 9698S) and TGF-β (1:1000; Cell Signaling Technology, 3711S). Protein bands were detected by electrochemiluminescence (ECL) using an Odyssey Imaging System (Li-Cor Biosciences, Lincoln, NE, USA), and analyzed using software ImageJ. Plasma TNF-α was determined using a mouse TNF-α Quantikine ELISA Kit (R&D Systems, Catalog# MTA00B, Minneapolis, MN, USA) together with a microplate reader (Biotek, Winooski, VT, USA).

### 2.7. Statistical Analysis

All data are expressed as means ± standard deviation (SD), and statistical differences determined by ANOVA with Bonferroni’s post hoc test or Tukey’s test for data analysis of three or multiple groups with normal distributions and equal variance, and non-parametric Kruskal-Wallis test with Dunn’s post hoc test for comparisons of multiple groups with normal distributions and unequal variance or abnormal distributions. All statistical comparisons were performed using GraphPad Prism 8.0 software (San Diego, CA, USA). A two-tailed *p* < 0.05 was considered statistically significant.

## 3. Results

### 3.1. NAC Treatment Improved Insulin Resistance without Significant Changes in Plasma Insulin Levels

**Figure 1:**
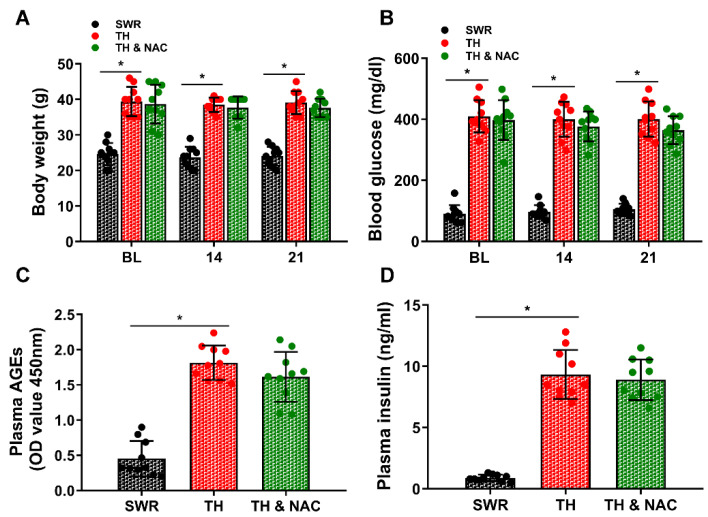
Body weight and the levels of blood glucose, plasma AGEs, and insulin in diabetic and non-diabetic mice. The body weight, blood glucose, and plasma AGEs levels were obtained before creation of limb ischemia in mice. Body weight (**A**), blood glucose (**B**), plasma AGEs (**C**), and insulin (**D**) were all significantly increased in diabetic TALLYHO/JngJ (TH) mice compared to the non-diabetic SWR/J (SWR) mice. No significant changes in the levels of blood glucose, plasma AGEs, and insulin were observed in diabetic TH mice with NAC treatment at day 21. AGEs, advanced glycation end products; BL, before ligation; NAC, N-acetylcysteine; TH, diabetic TALLYHO/JngJ mice with CLI operation; SWR, non-diabetic SWR/J mice with CLI operation; TH & NAC, TALLYHO/JngJ mice with CLI operation plus NAC treatment. All data are shown as means ± SD. * *p* < 0.01; *n* = 10/group. Statistical differences were determined by two-way repeated measures ANOVA followed by Tukey’s post hoc test (**A**,**B**) and one-way ANOVA with Tukey’s test (**C**,**D**).

**Figure 2:**
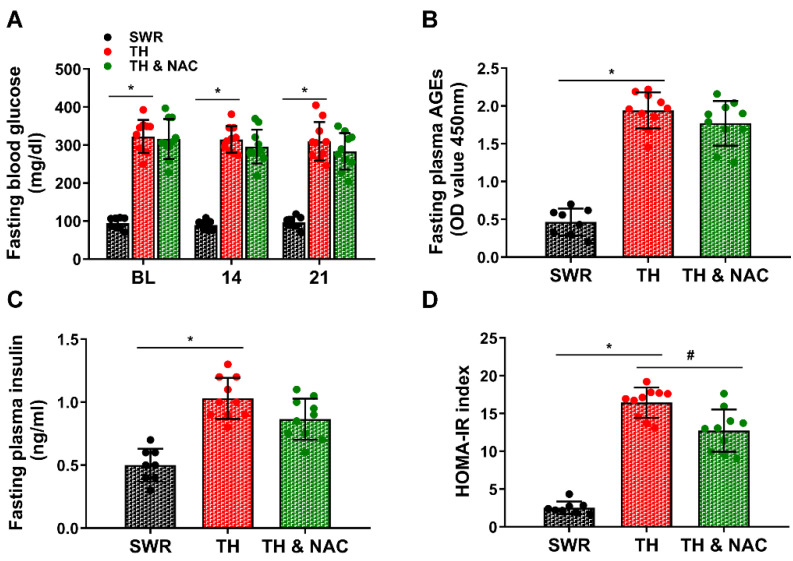
Fasting blood glucose, plasma AGEs, and insulin, and HOMA-IR index in diabetic and non-diabetic mice. Blood glucose, plasma AGEs, and insulin levels were measured after overnight fasting. The fasting blood glucose (**A**), plasma AGEs (**B**), plasma insulin (**C**), and HOMA-IR index (**D**) were significantly increased in diabetic TH mice compared to the non-diabetic SWR/J mice (* *p* < 0.01; *n* = 8–10/group). There were no significant changes in fasting blood glucose, plasma AGEs levels, and plasma insulin levels observed in diabetic TH mice with NAC treatment, although there was a trend of reduction in TH mice with NAC treatment at day 21. However, HOMA-IR index in TH mice with NAC treatment was significantly decreased compared to TH mice without NAC treatment (# *p* < 0.01; *n* = 8–10/group). HOMA-IR, homeostatic model assessment of insulin resistance; AGEs, advanced glycation end products; BL, before ligation; NAC, N-acetylcysteine; TH, diabetic TALLYHO/JngJ mice with CLI operation; SWR, non-diabetic SWR/J mice with CLI operation; TH & NAC, TALLYHO/JngJ mice with CLI operation plus NAC treatment. All data are shown as means ± SD. Statistical differences were determined by two-way ANOVA followed by Tukey’s test (**A**) and one-way ANOVA with Tukey’s test (**B**–**D**).

To confirm the suitability of the T2D mouse model, body weight and the levels of blood glucose, plasma AGEs, and plasma insulin were measured before and on days 14 and 21 after implementation of limb ischemia. As shown in Figure 1A–C, body weight, blood glucose, plasma AGEs, and plasma insulin levels were significantly increased in diabetic TALLYHO/JngJ (TH) mice compared to non-diabetic SWR/J (SWR) mice (* *p* < 0.01; *n* = 10/group). There were no significant changes in body weight, blood glucose, plasma AGEs, and plasma insulin levels observed in diabetic TH mice with or without NAC treatment (Figure 1B–D). The blood glucose, plasma AGEs, plasma insulin, and insulin resistance index were also evaluated after overnight fasting. There were no significant changes in fasting blood glucose, plasma AGEs, and plasma insulin levels observed in diabetic TH mice with NAC treatment although there was a trend of reduction in TH mice with NAC treatment at day 21. However, the calculated HOMA-IR index for NAC-treated TH mice was significantly decreased, compared to TH mice not treated with NAC (Figure 2, # *p* < 0.01; *n* = 8–10/group).

### 3.2. NAC Treatment Enhanced the Recovery of Blood Flow and Function of Diabetic Ischemic Limb

**Figure 3:**
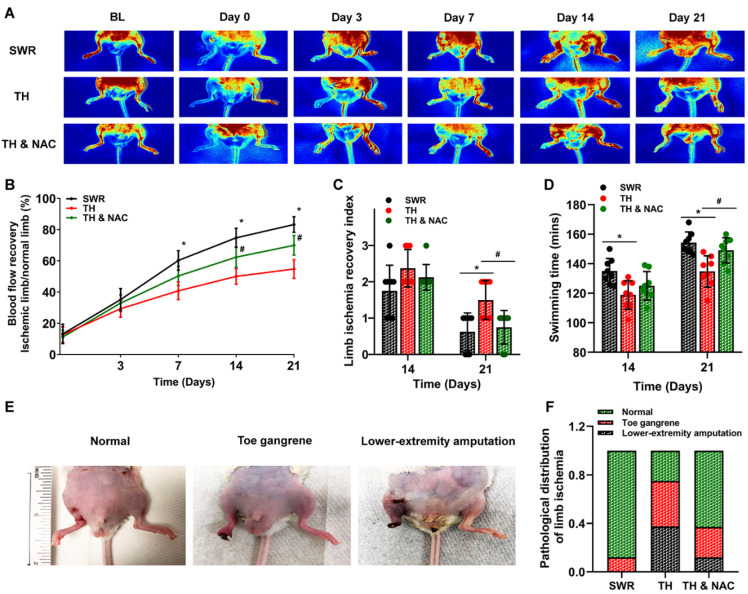
NAC treatment enhanced the recovery of blood flow and function of diabetic ischemic limb. Blood flow recovery as reflected by the ratio of ischemic limb (right hind limb)/non-ischemic limb (left hind limb) was significantly decreased in diabetic mice compared to non-diabetic mice at days 7, 14, and 21 after CLI (* *p* < 0.01, **A**,**B**). NAC treatment significantly enhanced the recovery of blood perfusion of diabetic ischemic limbs at days 14 and 21 after CLI (# *p* < 0.01; **A**,**B**). Function recovery of ischemic limb, as evaluated using a swimming endurance test and a semi-quantitative assessment of ambulatory impairment and limitation of the ischemic limb (modified clinical standard score), was also significantly decreased in diabetic mice compared to non-diabetic mice at days 14 and 21 (* *p* < 0.05; **C**,**D**). NAC treatment significantly enhanced functional recovery of diabetic ischemic limbs at day 21 (# *p* < 0.05; **C**,**D**). In addition, the rate of limb amputation was markedly increased in diabetic ischemic limb compared to the non-diabetic, and NAC treatment prevented the increase of limb amputation in diabetic ischemic limb (**E**,**F**). BL, before ligation; TH, diabetic TALLYHO/JngJ mice with CLI operation; SWR, non-diabetic SWR/J mice with CLI operation; TH & NAC, TALLYHO/JngJ mice with CLI operation plus NAC treatment. All data are shown as means ± SD. *n* = 8/group. Significant differences were determined with two-way repeated measures ANOVA followed by Tukey’s test.

Successful creation of CLI was confirmed using LDPI with no measurable signal for blood perfusion detected (Figure 3A). As demonstrated in Figure 3A,B, blood flow recovery, as reflected by the ratio of ischemic limb (right)/non-ischemic limb (left), was significantly decreased in diabetic mice compared to non-diabetic mice (TH vs. SWR 40.8% vs 60.3% at day 7, 50.0% vs 74.8% at day 14, and 54.8% vs 83.3% at day 21, respectively, * *p* < 0.01). Continuous NAC treatment significantly enhanced the recovery of blood perfusion of diabetic ischemic limb at day 14 and 21 (TH vs. TH & NAC 50.0% vs. 62.4% at day 14, and 54.8% vs. 70.0% at day 21, respectively, # *p* < 0.01).

As shown in Figure 3C,D, functional recovery of the ischemic limb, evaluated using a swimming endurance test and a semi-quantitative assessment of ambulatory impairment and limitation of the ischemic limb (modified clinical standard score), was also significantly decreased in diabetic mice compared to non-diabetic mice (for swimming time: TH vs. SWR 118 min vs. 135 min at day 14, and 135 min vs. 154 min at day 21; for limb ischemia recovery index: TH vs. SWR 1.5 vs. 0.6 at day 21; * *p* < 0.05). NAC treatment significantly enhanced recovery of function in diabetic ischemic limbs (swimming time: TH vs. TH & NAC 135 min vs 149 min at day 21; limb ischemia recovery index: TH vs. TH & NAC 1.5 vs 0.8 at day 21; # *p* < 0.05). In addition, the rate of lower-extremity amputation was markedly increased in diabetic ischemic limb compared to the non-diabetic (TH vs. SWR 37.5% vs. 0%), and continuous NAC treatment significantly decreased the rate of lower-extremity amputation in diabetic ischemic limb (TH vs. TH & NAC 37.5% vs. 12.0%; Figure 3E). The distribution of normal, toe gangrene, and lower-extremity amputation in each group is summarized in Figure 3F.

### 3.3. NAC Treatment Improved Capillary Density and Muscular Structure, While Decreasing Inflammation and Fibrosis in Diabetic Ischemic Limbs

**Figure 4:**
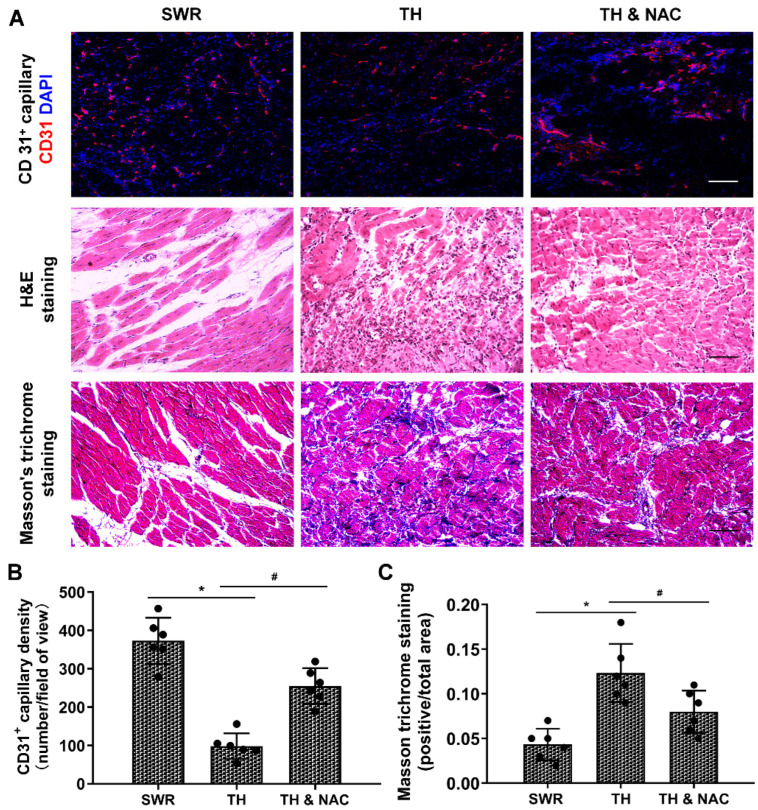
NAC treatment improved CD31^+^ capillary density and preserved muscular structure in diabetic ischemic limb. Ischemic muscle tissues were harvested at day 21 after CLI for histological analysis. CD31^+^ capillary density (**A**) was significantly decreased with disorganized muscular structure (**B**) and increased tissue fibrosis (**C**) in diabetic ischemic limb compared to the controls (* *p* < 0.01). NAC treatment significantly prevented the reduction of CD31^+^ capillary density and preserved the morphology and structure of muscle with decreased inflammatory infiltration and tissue fibrosis (# *p* < 0.05; **B**,**C**). DAPI, 4′,6-diamidino-2-phenylindole; TH, diabetic TALLYHO/JngJ mice with CLI operation; SWR, non-diabetic SWR/J mice with CLI operation; TH & NAC, TALLYHO/JngJ mice with CLI operation plus NAC treatment. Scale bar, 50 μm. All data are shown as means ± SD. *n* = 6/group. Significant difference was determined with one-way ANOVA with Bonferroni’s post hoc test.

Gastrocnemius muscle samples of the ischemic limb were harvested at day 21 after CLI for ex vivo histological evaluation for capillary density, muscular structure, inflammatory infiltration, and fibrosis. As shown in Figure 4A–C, CD31^+^ capillary density was significantly decreased in the diabetic ischemic limb with disorganized muscular structure and increased tissue fibrosis compared to the control (for CD31^+^ capillary density: TH vs. SWR 98.5 vs. 373.0; for fibrosis area: TH vs. SWR 12.3% vs. 5.2%; * *p* < 0.01). NAC treatment effectively preserved CD31^+^ capillary density and the structure of muscle, and significantly decreased inflammatory infiltration and tissue fibrosis (for CD31^+^ capillary density: TH vs.TH & NAC 98.5 vs. 255.0; for fibrosis area: TH vs. TH & NAC 12.3% vs. 8.0%; # *p* < 0.05). In addition, the expression of VEGFR2 was markedly decreased in the ischemic muscle tissue of diabetic mice, while significantly increased TGF-β levels were noted as compared to non-diabetic controls. NAC treatment partially reversed the changes of VEGFR2 and TGF-β levels in diabetic ischemic limbs (Figure S1, # *p* < 0.05).

### 3.4. NAC Effectively Prevented ROS Production, While Increasing the Expression of Antioxidant Enzymes in Diabetic Ischemic Limbs

**Figure 5:**
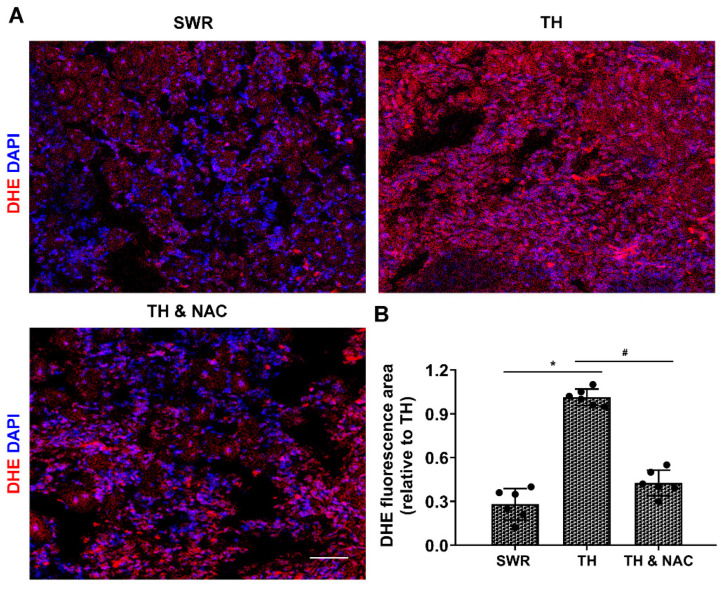
NAC treatment prevented ROS overproduction in diabetic ischemic limb. Detection of in situ ROS production was performed both in the ischemic limb of diabetic and non-diabetic mice using DHE staining. Tissue ROS levels in diabetic ischemic limb were significantly increased compared to the non-diabetic ischemic limb at day 21 after CLI (TH versus SWR 1.01/0.28, * *p* < 0.01, **A**,**B**), whereas NAC treatment significantly prevented the increase of ROS production in diabetic ischemic limbs (TH versus TH & NAC 1.01/0.43, # *p* < 0.01, **A**,**B**). DAPI, 4′,6-diamidino-2-phenylindole; DHE, fluorescent dye dihydroethidium; TH, diabetic TALLYHO/JngJ mice with CLI operation; SWR, non-diabetic SWR/J mice with CLI operation; TH & NAC, TALLYHO/JngJ mice with CLI operation plus NAC treatment. Scale bar, 50 μm. All data are shown as means ± SD; *n* = 6/group. Significant differences were determined with one-way ANOVA with Bonferroni’s post hoc test.

**Figure 6:**
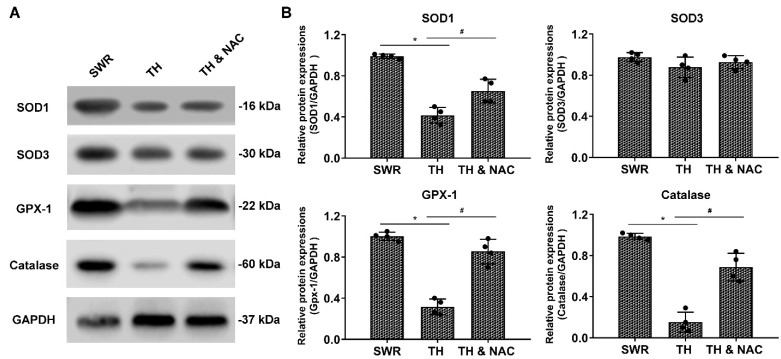
NAC preserved the expression of antioxidant enzymes in ischemic muscle in type-2 diabetic mice. To exam the effect of NAC on the expression of antioxidant enzymes in diabetic ischemic limbs, ischemic muscle tissue was harvested for Western blot analysis. As demonstrated in panels (**A**,**B**), the expression of SOD-1, Gpx-1, and catalase were significantly decreased in diabetic ischemic limbs compared to non-diabetic control (*n* = 4, * *p* < 0.05 for TH versus SWR); there was a trend towards a decreased expression of SOD-3, but it was not significant statistically. NAC treatment significantly prevented the reduction in the expression of SOD-1, Gpx-1, and catalase in diabetic ischemic limb muscle tissue (*n* = 4, # *p* < 0.05 for TH versus TH & NAC). SOD-1, copper-zine superoxide dismutase in cytoplasm; SOD-3, extracellular SOD; Gpx-1, glutathione peroxidase-1; GAPDH, glyceraldehyde 3-phosphate dehydrogenase; TH, diabetic TALLYHO/JngJ mice with CLI operation; SWR, non-diabetic SWR/J mice with CLI operation; TH & NAC, TALLYHO/JngJ mice with CLI operation plus NAC treatment. All data are shown as means ± SD. Significant differences were determined with one-way ANOVA with Bonferroni’s post hoc test or Kruskal-Wallis test with Dunn post hoc test.

ROS production was measured in the ischemic limbs of diabetic and non-diabetic mice using DHE staining. Tissue ROS level in diabetic ischemic limbs was significantly increased by almost four-fold over that in non-diabetic ischemic limbs at day 21 after CLI (TH vs. SWR 1.01 vs. 0.28, * *p* < 0.01). NAC treatment significantly prevented the increase of ROS production in diabetic ischemic limbs (TH vs. TH & NAC 1.01 vs. 0.43, # *p* < 0.01, Figure 5A,B). To determine the effect of NAC on levels of antioxidant enzymes in diabetic ischemic limbs, ischemic muscle tissue was harvested for immunoblot analysis. As demonstrated in Figure 6A,B, the expression of SOD-1, Gpx-1, and catalase were significantly decreased in diabetic ischemic limbs compared to non-diabetic controls (* *p* < 0.05). NAC treatment significantly preserved the expression of SOD-1, Gpx-1, and catalase in diabetic ischemic limbs (# *p* < 0.05).

### 3.5. NAC Treatment Increased the Phosphorylation of Akt and eNOS Expression, While Decreasing Plasma TNF-a and Phosphorylated IRS-1 in Diabetic Ischemic Limbs

**Figure 7:**
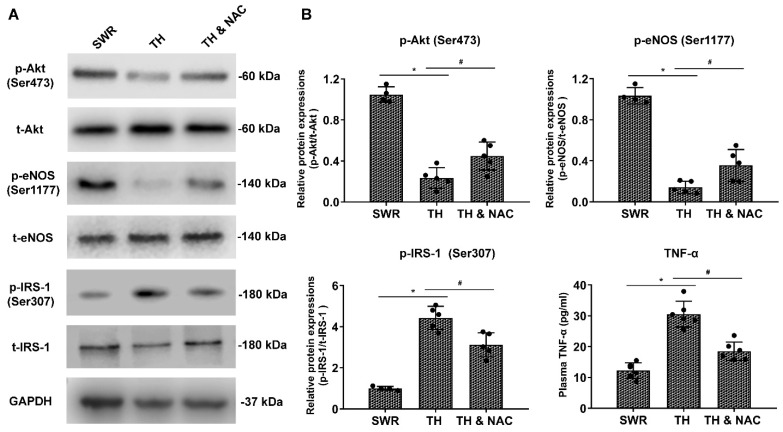
NAC treatment increased the phosphorylation of Akt (Ser473) and eNOS (Ser1177) expression, while decreasing plasma TNF-a and phosphorylated IRS-1 (Ser307) expression in diabetic ischemic limbs. As shown in panels (**A**,**B**), levels of p-IRS-1 and TNF-α were significantly increased together with a decrease in total IRS-1 compared to those of non-diabetic controls. NAC treatment partially, but significantly, reversed the changes in p-IRS-1 and TNF-α (*n* = 4–6, # *p* < 0.05 for TH versus TH & NAC, * *p* < 0.01 for TH versus SWR). In addition, the expression of p-Akt and p-eNOS was markedly decreased in the ischemic muscle tissue of diabetic mice without apparent changes in either total Akt or eNOS levels. NAC treatment significantly improved the levels of p-Akt and p-eNOS in diabetic ischemic limbs (*n* = 4–6, # *p* < 0.05 for TH versus TH & NAC, * *p* < 0.01 for TH versus SWR). IRS-1, insulin receptor substrate 1; GAPDH, glyceraldehyde 3-phosphate dehydrogenase; TH, diabetic TALLYHO/JngJ mice with CLI operation; SWR, non-diabetic SWR/J mice with CLI operation; TH & NAC, TALLYHO/JngJ mice with CLI operation plus NAC treatment. All data are shown as means ± SD. Significant differences were determined with one-way ANOVA with Bonferroni’s post hoc test or Kruskal-Wallis test with Dunn post hoc test.

As indicators of protein activity, the levels of phosphorylated IRS-1 (Ser307), Akt (Ser473), and eNOS (Ser1177) in ischemic muscle tissue, as well as TNF-α in plasma were evaluated using Western blot and ELISA analysis, respectively. As shown in Figure 7A,B, levels of p-IRS-1 (Ser307) and TNF-α were significantly increased together with a decrease in total IRS-1 compared to those of non-diabetic controls. NAC treatment partially, but significantly, reversed the changes in p-IRS-1 (Ser307) and TNF-α (# *p* < 0.05). In addition, the expression of p-Akt (Ser473) and p-eNOS (Ser1177) was markedly decreased in the ischemic muscle tissue of diabetic mice without apparent changes in either total Akt or eNOS levels. NAC treatment significantly improved the levels of p-Akt (Ser473) and p-eNOS (Ser1177) in diabetic ischemic limbs (# *p* < 0.05).

## 4. Discussion

In the present study, we demonstrated a significant increase in ROS formation in the diabetic ischemic hindlimb, associated with impaired recovery of blood flow and mechanical function. Compared to non-diabetic controls, levels of antioxidant enzyme expression including SOD-1, Gpx-1, and catalase were significantly decreased. Further, phosphorylated Akt (Ser473) and eNOS (Ser1177) levels were significantly decreased in the diabetic ischemic limb, while levels of phosphorylated IRS-1 (Ser307) and plasma TNF-α were significantly increased. NAC treatment in T2D mice effectively preserved the expression of the antioxidant enzymes (SOD-1, Gpx-1, and catalase), while attenuating ROS production in the diabetic ischemic limb together with reversed levels of phosphorylated IRS-1, Akt, and eNOS, and plasma TNF-a. Associated with these changes in signaling was improved post-ischemia recovery of blood perfusion and limb function in T2D mice with increased capillary density, as well as decreases in inflammatory cell infiltration and rate of lower-extremity amputation. Collectively, these data demonstrate, for the first time, that NAC treatment significantly improves both structural and functional recovery following induction of CLI in a translationally relevant model of T2D.

DM is an important risk factor for major cardiovascular diseases, including severe PAD with CLI and non-healing chronic ulcers without effective treatments [33]. NAC was reported to prevent ischemia-induced ROS formation and oxidative stress and improve limb recovery following ischemic injury [34,35]. Other potential mechanisms include promoting arterial thrombolysis [35], preserving endothelial function [36,37], and maintaining populations of circulating endothelial progenitor cells [16]. In the setting of diabetes, NAC was reported to improve glucose homeostasis in mouse models of insulin resistance secondary to adipocyte insulin receptor deletion and obesity [38], to attenuate ischemic injury in diabetic heart by restoring caveolin-3/eNOS signaling [15], and to protect the brain against ischemia-reperfusion injury in streptozotocin-induced and genetic Akita mouse models of Type 1 diabetes [39]. In the present study, we observed that NAC treatment significantly enhanced the recovery of blood perfusion and function of ischemic limbs in T2D. Our data showed that increased ROS production was associated with decreased activation of Akt and eNOS, as well as decreased VEGFR2 expression in diabetic ischemic muscle tissue. NAC treatment significantly decreased ROS production in the ischemic muscle tissue with partial restoration of phosphorylated Akt and eNOS, and VEGFR2 expression.

Sustained hyperglycaemia accelerates the formation of AGEs in diabetes, leading to excess ROS production and tissue inflammation via activation of RAGE signaling cascade [40]. In addition, increased superoxide production could further increase the formation of AGEs and enhance the expression of AGE receptors facilitating interaction with its ligands, ultimately activating proinflammatory pathways with long-lasting epigenetic changes [7]. In the present study, as expected we observed that diabetic mice had significantly increased levels of blood glucose and plasma AGEs. ROS production was significantly increased in the diabetic ischemic limb in association with decreased expression of antioxidant enzymes including SOD-1, Gpx-1, and catalase in muscle tissue. Increased ROS production in the diabetic ischemic limb may result from the combined increase in AGEs levels and decrease in expression of antioxidant enzymes in muscle tissue. NAC treatment significantly decreased ROS production in diabetic ischemic muscles with partially preserved expression of antioxidant enzymes SOD-1, Gpx-1, and catalase. Of note, NAC was reported to decrease AGEs levels and ameliorate AGEs-induced effects [41,42,43]. However, there was no significant change in plasma AGEs levels in the present study. This suggests that, within the context of this study, NAC improved redox and inflammatory status in the presence of already formed AGEs, while perhaps inhibiting the formation of additional AGEs.

Increased oxidative stress has been shown to alter extracellular matrix structure and function [44]. Fibrosis is a part of long-term changes following significant ischemic injury especially in muscles. TGF-β1 signaling, metabolic abnormalities, and chronic low-grade inflammation are believed to be important in the pathogenesis of fibrosis [45,46]. However, oxidative stress and anti-oxidative mechanisms may also be critical to the development and progression of fibrosis [46]. DM is associated with increased fibrosis in many organs/tissues, including heart, liver, kidney, and bladder, contributing to organ/tissue dysfunction [47,48,49,50]. However, the effect of DM on tissue fibrosis following limb ischemia has not been defined. The data from the present study showed that tissue fibrosis in the ischemic limb muscle was significantly increased in T2D in association with a substantial increase in ROS formation and TGF-β expression. NAC treatment effectively suppressed excessive ROS production, partially attenuated TGF-β expression, and significantly decreased tissue fibrosis in the ischemic limb muscle in T2D mice without significant changes in the levels of plasma AGEs and blood glucose. These data suggest that the interaction between ischemia-induced ROS production and TGF-β expression may play an important role in fibrosis in diabetic ischemic limb that needs further investigations.

Insulin mediates energy uptake through inhibiting hepatic glucose production and increasing glucose uptake into peripheral sites such as muscle and adipose tissue [51]. The insulin receptor and IRS proteins are regulated by a number of mechanisms including degradation, differential expression, and post-translational modification by serine/tyrosine phosphorylation [52,53,54,55]. Impaired insulin responsiveness/insulin resistance is commonly linked to serine phosphorylation of IRS-1 [56]; phosphorylation of IRS-1 on Ser307 (human Ser312) results in its dissociation from the insulin receptor, and also impairs insulin downstream signaling [57,58]. Oxidative stress has been considered to be a major hallmark of the pathogenesis and development of major complications in T2D [59]. Excessive ROS production can also significantly impair glucose homeostasis and increase insulin resistance, leading to decreased efficacy of hypoglycemic drugs [60,61]. The present study showed that the levels of ROS and TNF-α were significantly increased in the diabetic ischemic limb compared to those of non-diabetic controls. Levels of p-IRS-1 (Ser307) and the HOMA-IR index were also substantially increased since the deleterious effects of oxidative stress and ROS production in diabetic ischemic limb. Importantly, NAC treatment was observed to decrease the levels of ischemic ROS production and plasma TNF-α, together with a partial, yet significant, reversal of the changes of p-IRS-1 (Ser307) and p-AKT (Ser473) and the HOMA-IR index. These data suggest that long-term NAC treatment may ameliorate impaired insulin signaling by p-IRS-1 on Ser307 in an ROS-dependent manner in ischemic tissue in T2D.

In the present study, NAC enhanced the recovery of blood perfusion and function in the ischemic limb of T2D mice without significant changes in blood glucose and AGE levels. However, NAC has been shown to attenuate AGEs- and high glucose-mediated ROS production and apoptosis in endothelial cells [62,63,64]. Of further relevance, eNOS is critical to endothelial function, and oxidative stress can cause both dysregulation of eNOS and endothelial dysfunction [65]. NAC administration has previously been shown to increase NO bioavailability [66], and decrease the endothelial activation [67] in T2D. It could be important and interesting to determine if other antioxidants like SOD mimic Tempol or ROS scavengers or natural and synthetic antioxidants could have similar effect and outcomes on the recovery of diabetic critical limb ischemia as NAC [68].

NAC was previously reported to attenuate inflammatory and oxidative damages and improve limb function in a non-diabetic murine CLI model [16,34,69]. NAC has also been shown to have the ameliorative potential against a number of diabetes-associated complications, including diabetes-induced cardiac damage through inhibition of oxidative stress in preclinical studies as highlighted in a recent review [70]. The findings from the present study that NAC treatment provided significant improvement on the recovery of blood flow and function of critical limb ischemia in T2D mice have substantial translational values and clinical significance. Over 90% of diabetic patients have T2D, and a significant portion of diabetic patients develop significant limb ischemia and unhealing skin ulcers without effective treatment. NAC is an FDA-approved generic drug with an established safety record and ready to be used in patients for prevention and/or treatment of diabetic CLI. Therefore, a large clinical study is needed to determine if NAC could provide similar benefit for diabetic patients with significant limb ischemia as in the mouse model.

There were limitations in the present study, including: (1) only male mice were used. It is important to determine if the protective effect of NAC on diabetic CLI could be present in females as well. (2) Only one dose was used in the present study. It is important to demonstrate if there is a dose-dependent effect of NAC on diabetic CLI. (3) NAC was placed in the drinking water in the present study. It is highly possible that each mouse might get different amount of NAC. (4) Diabetic CLI usually occurs in patients with advanced age. Thus, it is important to determine if NAC could improve the outcome of diabetic CLI in aging diabetic mice.

## 5. Conclusions

**Figure 8:**
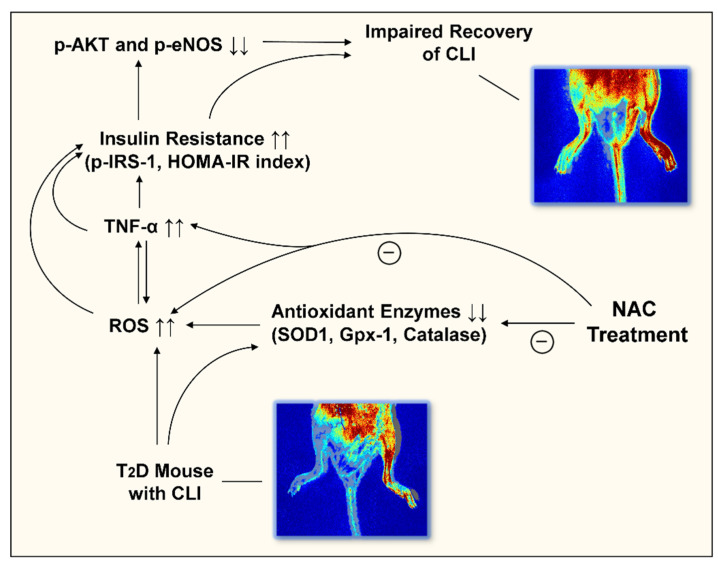
Illustration of potential mechanisms for the beneficial effects of NAC on the recovery of CLI in T2D mice. We proposed that NAC enhances the recovery of CLI in T2D mice through multiple mechanisms: (1) attenuation of excessive ROS production; (2) reduction of plasma TNF-α level; (3) decrease of insulin resistance (evidenced by increased levels of p-IRS-1 and the HOMA-IR index) in ischemic limbs; and (4) preservation of the expressions of antioxidant enzymes, phosphorylated IRS-1, Akt, and eNOS. CLI, Critical limb ischemia; T2D, type-2 diabetes; NAC, N-acetylcysteine; ROS, reactive oxygen species; SOD-1, copper-zine superoxide dismutase in cytoplasm; Gpx-1, glutathione peroxidase-1; HOMA-IR, homeostatic model assessment of insulin resistance; -, inhibit; ↑, increase; ↓, decrease.

In summary, the present study demonstrated that NAC treatment enhanced the recovery of blood flow and function following ischemia in T2D in association with attenuated ROS production and plasma TNF-α level with preserved expressions of antioxidant enzymes, and partially reversed the levels of phosphorylated IRS-1, Akt, and eNOS as shown in Figure 8, thus improving redox/inflammatory status and insulin resistance in diabetic ischemic tissue. The findings may provide evidence in support of NAC being considered as a potential therapeutic or adjuvant therapeutic option for patients with diabetes and severe limb ischemia. Future clinical studies are needed to confirm the findings of the present study in patients with T2D and CLI. Further studies are also needed to investigate the effect of NAC in combination with current standard medical regiments on the outcomes of diabetic CLI to ensure clinically relevant translation.

## Data Availability

All of the data is contained within the article and the supplementary materials.

## Supplementary Materials

The following supporting information can be downloaded at: https://www.mdpi.com/article/10.3390/antiox11061097/s1, Figure S1: NAC treatment increased VEGFR2 expression, and decreased TGF-β expression in diabetic ischemic limbs.
