# Supplementary material for: N-Acetylcysteine Enhances the Recovery of Ischemic Limb in Type-2 Diabetic Mice
Source: Antioxidants (Basel). 2022 May 31;11(6):1097. doi: 10.3390/antiox11061097 (PMC9219773; doi:10.3390/antiox11061097)
Supplement: Supplementary file 1 [file antioxidants-11-01097-s001.zip › antioxidants-1680589-supplementary.pdf]

Supplementary Figures and Figure Legends

Figure S1

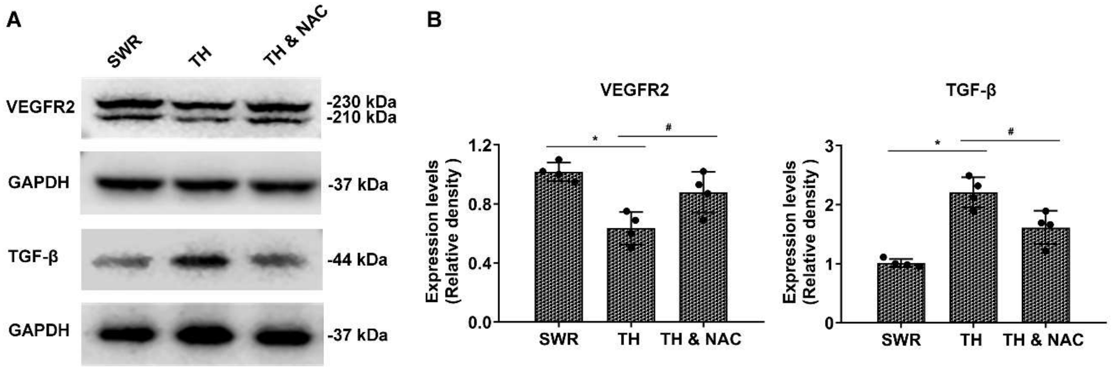

Fig. S1. NAC treatment increased VEGFR2 expression, and decreased TGF-β expression in diabetic ischemic limbs. Gastrocnemius muscle tissue of ischemic limbs were harvested for detection of angiogenesis- and fibrosis-related proteins using Western blot assay. As shown in panels **A-B,** the expression of VEGFR2 in ischemic muscle tissue in diabetic mice was markedly decreased with increased levels of TGF-β as compared to non-diabetic controls. NAC treatment partially, yet significantly, reversed the changes of VEGFR2 and TGF-β levels in diabetic ischemic limbs (n=4, #$p<0.05$ for TH versus TH & NAC, C-D). VEGFR2, vascular endothelial growth factor receptor 2; TGF-β, transforming growth factor beta; GAPDH, glyceraldehyde 3-phosphate dehydrogenase; other abbreviations as in Figure 2. All data were shown as means ± SD. Significant difference was determined with one-way ANOVA with Bonferroni's post hoc test or Kruskal-Wallis test with Dunn post hoc test.
